# Intravital longitudinal wide-area imaging of dynamic bone marrow engraftment and multilineage differentiation through nuclear-cytoplasmic labeling

**DOI:** 10.1371/journal.pone.0187660

**Published:** 2017-11-03

**Authors:** Soyeon Ahn, Kibaek Choe, Seunghun Lee, Kangsan Kim, Eunjoo Song, Howon Seo, Injune Kim, Pilhan Kim

**Affiliations:** 1 Graduate School of Nanoscience and Technology, Korea Advanced Institute of Science and Technology, Daejeon, Republic of Korea; 2 KI for Health Science and Technology (KIHST), Korea Advanced Institute of Science and Technology, Daejeon, Republic of Korea; 3 Graduate School of Medical Science and Engineering, Korea Advanced Institute of Science and Technology, Daejeon, Republic of Korea; Fraunhofer Research Institution of Marine Biotechnology, GERMANY

## Abstract

Bone marrow is a vital tissue that produces the majority of erythrocytes, thrombocytes, and immune cells. Bone marrow transplantation (BMT) has been widely performed in patients with blood disorders and cancers. However, the cellular-level behaviors of the transplanted bone marrow cells over wide-areas of the host bone marrow after the BMT are not fully understood yet. In this work, we performed a longitudinal wide-area cellular-level observation of the calvarial bone marrow after the BMT *in vivo*. Using a H2B-GFP/β-actin-DsRed double-transgenic mouse model as a donor, a subcellular-level nuclear-cytoplasmic visualization of the transplanted bone marrow cells was achieved, which enabled a direct *in vivo* dynamic monitoring of the distribution and proliferation of the transplanted bone marrow cells. The same spots in the wide-area of the calvarial bone marrow were repeatedly identified using fluorescently labeled vasculature as a distinct landmark. It revealed various dynamic cellular-level behaviors of the transplanted BM cells in early stage such as cluster formation, migration, and active proliferation *in vivo*.

## Introduction

Bone marrow (BM) inside the bone is a soft flexible tissue that produces the majority of erythrocytes, thrombocytes, and immune cells. Every day, approximately 200 billion blood cells are newly produced in the human bone marrow. As the source of this massive cellular production, bone marrow houses hematopoietic stem cells (HSCs) that can differentiate into various kinds of blood cells such as erythrocytes, thrombocytes, and immune cells including neutrophils and lymphocytes [1–3]. Due to its critical role in the homeostasis of the circulatory and immune cellular systems, HSC has been widely used in various clinical situations, mainly in the form of bone marrow transplantation (BMT) [4, 5]. BMT has been performed in patients with blood cancer or blood cell disorders such as leukemia, anemia, and multiple myeloma. In detail, donor bone marrow cells including HSC and hematopoietic progenitor cells were expanded *in vitro* by supplementing various growth factors including granulocyte colony stimulating factors and then transferred to a recipient by blood transfusion. The transferred HSCs efficiently home to the bone marrow and gradually reconstitute the whole hematopoietic system of the recipient [4–6].

However, despite its frequent medical practices, how the transferred BM cells including stem cells and progenitors behave inside the bone marrow of the recipient at early time point after the BMT is not well understood yet [7–10]. Especially, our knowledge on the cellular-level behaviors of the transplanted BM cells which dynamically change over time after the BMT is relatively limited, which has been one of the limiting factors in previous efforts to improve the efficacy of BMT and to reduce the detrimental side effects [11, 12]. Recently, there have been several studies on *in vivo* microscopy imaging of bone marrow including optical coherence tomography (OCT) [13], two-photon excited fluorescence (TPEF) microscopy and confocal microscopy [14–21], all of which can be promising methods to monitor the dynamic behavior of transplanted BM cells. Previous intravital cellular-level imaging studies on calvarial BM has mostly focused on the cellular behaviors of the transplanted BM cells in a relatively small microscopic area of the BM, such as the localization of HSCs to the vasculature with distinct molecular expression [18], or a specific niche such as the endosteal surface [22]. Dynamically changing cellular-level behaviors of the transplanted BM cells in a wide area of the calvarial BM have remained relatively unknown.

In this work, we performed *in vivo* longitudinal wide-area visualization of various cellular-level behaviors of the transplanted bone marrow cells in calvarial bone marrow with a custom-built video-rate laser-scanning confocal microscopy system [23–26]. Notably, with a genetically engineered histone H2B-GFP/β-actin-DsRed double-transgenic mouse [24] as the donor mouse for syngeneic BMT in which individual cells express GFP exclusively in the nucleus and DsRed in both the cytoplasm and the nucleus, we achieved subcellular-level nuclear-cytoplasmic morphological visualization of the transplanted bone marrow cells [27–29] in the calvarial bone marrow of a recipient mouse *in vivo*. It enabled a dynamic *in vivo* visualization of migration, proliferation and differentiation of the transplanted bone marrow cells. At the same time, bone marrow vasculature was also visualized by fluorescently labeling the endothelial cells. By using the distinct vasculature as a landmark along with a stereotaxic mount set-up, we successfully achieved repetitive imaging of the same spots in the bone marrow over several days. Longitudinal visualization clearly revealed the dynamic cellular-level behaviors of the transplanted BM cells including cluster formation, migration, active proliferation, and differentiation into certain cell types *in vivo*.

## Materials and methods

### Animal model

All animal experiments were performed in accordance with the standard guidelines for the care and use of laboratory animals and were approved by the Institutional Animal Care and Use Committee (IACUC) of KAIST (protocol no. KA2013-10). All surgeries were performed under anesthesia, and all efforts were made to minimize suffering. Mice were individually housed in ventilated and temperature & humidity-controlled cages (22.5°C, 52.5%) under 12:12h light:dark cycle and provided with standard diet and water *ad libitum*. A histone H2B-eGFP/β-actin-DsRed double-transgenic mouse (C57BL/6 strain) was generated by cross-breeding H2B-GFP transgenic mice ([30], Stock number; 006069, Jackson Laboratory) and β-actin-DsRed transgenic mice (generously provided from Dr. Koh at KAIST).

### Bone marrow transplantation

For syngeneic bone marrow transplantation, sixteen histone H2B-GFP/β-actin-DsRed double-transgenic mice (20–35 g body weight; 12–25 weeks, C57BL/6) were used as donors, and eight wildtype C57BL/6 mice (20–35 g; 12–20 weeks) were used as recipients. For harvesting bone marrow cells, donor mice were euthanized with CO_2_ gas in a euthanasia chamber. From the bone marrow cells harvested from the femur and tibia of the donor, c-kit^+^ bone marrow cells were isolated by Magnetic-activated cell sorting (MACS). To purify the c-kit^+^ cells, we used anti-mouse CD117 (c-kit) antibody conjugated with Biotin (13–1171, eBioscience) and anti-Biotin microbeads for MACS (130-090-485, Miltenyi Biotec) and performed automated MACS cell separation with an autoMACS pro separator (Miltenyi Biotec). 1 x 10^7^ purified c-kit^+^ cells were intravenously injected into the recipient mouse anesthetized with anesthesia drug (ketamine (70 mg/kg)—xylazine (10 mg/kg) mixture) after the sub-lethal irradiation of a dose of 6 Gy. Recipient animals underwent intravital imaging experiment under anesthesia on 1–4 days post transplantation. After experiment, post-surgical health monitoring of vital signs and body weight was performed daily for a minimum of seven days to ensure the post-operative recovery into pre-operative status. After the monitoring, recipient mice were euthanized with CO_2_ gas in a euthanasia chamber.

### Imaging system

To visualize the *in vivo* longitudinal dynamics of the transplanted bone marrow cells in the calvarial bone marrow, a custom-built video-rate laser-scanning confocal microscopy system [23–26] was used. Schematic diagram of the imaging system and a photograph of the mouse showing the imaging of the calvarial bone marrow are given in S1A Fig. Three continuous laser modules whose wavelengths were at 488 nm (MLD488, Cobolt), 561 nm (Jive, Cobolt), and 640 nm (MLD640, Cobolt) were used as excitation light sources for three-color fluorescence imaging. Three-color laser beams were collinearly combined by dichroic beam splitters (DBS1; FF593-Di03, DBS2; FF520-Di02, Semrock) and delivered to the laser-beam scanner by a multi-edge dichroic beam splitter (DBS3; Di01-R405/488/561/635, Semrock). The laser scanning mirrors were composed of a rotating polygonal mirror with 36 facets (MC-5, aluminum coated, Lincoln Laser) for x-axis scanning and a galvanometer scanning mirror (6230H, Cambridge Technology) for y-axis scanning. The two-dimensional raster-scanning laser beam was delivered to the commercial objective lens (LUCPlanFLN, 40X, NA 0.6, Olympus), providing imaging field of view of 250 μm x 250 μm. The fluorescence signals emitted from the calvarial bone marrow of the anesthetized mouse on the XYZ translational 3D stage (3DMS, Sutter Instrument) were epi-detected by the objective lens. De-scanned three-color fluorescence signals were spectrally divided by dichroic beam splitter (DBS4; FF560-Di01, DBS5; FF649-Di01, Semrock) and then detected by photomultiplier tubes (PMT; R9110, Hamamatsu) with bandpass filters (BPF1; FF02-525/50, BPF2; FF01-600/37, BPF3; FF01-685/40, Semrock). The voltage outputs of each PMT were digitalized by a 3-channel frame grabber (Solios, Matrox) with an 8-bit resolution at a sampling rate of 10 MHz. With custom-written imaging software based on the Matrox Imaging Library (MIL9, Matrox) and Visual C^++^, video-rate movies were displayed and recorded in real time at a frame rate of 30 Hz and a frame size of 512 x 512 pixels.

### Stereotaxic mount setup

To hold the mouse cranium in a fixed position during repetitive imaging experiments, a modified stereotaxic mount set-up was implemented. The stereotaxic instruments consisted of ear bars with probe holders, which could control the latero-lateral axis of the skull, and a mouth adapter fixing the mouse incisive teeth which could control the rostro-caudal axis (S1B Fig). A U-shape holder attached to a compact kinematic mirror mount (KMS, Thorlabs) was used to place a cover-glass over the imaging area on the mouse cranium. The implemented stereotaxic mount set-up was assembled on a 3D translational stage for an anesthetized mouse as shown in S1C Fig. It enabled us to locate the same regions in the calvarial bone marrow with a distinctive anatomical feature of the cranium enabling repetitive imaging of the same area for longitudinal analysis.

### In vivo bone marrow imaging

Calvarial bone marrow has been widely used as an *in vivo* longitudinal imaging site [22, 31, 32] because the mouse cranium is easily accessible with a minimal scalp incision and thin enough to obtain relatively clear images from the inner bone marrow without further manipulation such as surgical thinning [33]. For intravital imaging, a mouse was anesthetized by intraperitoneal injection of a ketamine (70 mg/kg) and xylazine (10 mg/kg) cocktail mixture. The scalp was shaved by a hair clipper and depilatory cream and cleaned with alcohol swab. The cranium was exposed with a scalp incision and fixed with the stereotaxic mount stage (S1C Fig). Transplanted bone marrow cells were fluorescently detected by H2B-GFP expression in nuclei and β-actin-DsRed expression. Three hours before the imaging, blood vessels in the calvarial bone marrow were fluorescently labeled by intravenous injection of anti-CD31 monoclonal antibody (553370, BD Bioscience) conjugated with a far-red fluorophore, Alexa Fluor 647 (A-20186, Invitrogen) [34–37]. During imaging, the mouse body core temperature was maintained at 36°C by temperature monitoring with a rectal probe and a homeothermic control system (RightTemp, Kent Scientific) and warmed saline was topically applied to the exposed cranium every 10 minutes to avoid tissue drying. The depths of anesthesia was monitored during experiment by toe pinch and maintained with intraperitoneal injection of 12mg/kg of ketamine—xylazine cocktail mixture for boosting. After imaging, antiseptic was applied to the mouse scalp, and then, it was stitched up by a nylon suture (SK617, AILEE). Mice were placed in a clean cage for recovering from the anesthesia and monitored for signs of pain or distress until awake. To avoid an excessive inflammatory response during the repetitive imaging over several days, dexamethasone sodium phosphate (1.5 μg/g, Sigma-Aldrich) was administered once by intramuscular injection and carprofen (0.15 mg, Rimadyl, Pfizer) daily by intraperitoneal injection, and drinking water was supplied that contained sulfamethoxazole and trimethoprim (95 mg/kg/24 hours, each 40 mg/ml and 8 mg/ml in drinking water, Sigma-Aldrich) [38].

### Flow cytometry analysis

Harvested BM cells from the femur and tibia were resuspended in PBS containing 2% FBS, incubated at 4°C for 30–40 min. with the following antibodies: V450-conjugated anti-CD45 (BD Bioscience), APC-conjugated anti-CD11b (BD Bioscience), V450-conjugated anti-CD3e (BD Bioscience) and APC-conjugated anti-B220 (BD Bioscience). After the antibody incubation, cells were washed, centrifuged at 1500 rpm for 5 min. and resuspended in PBS containing 2% FBS. Analysis and cell sorting were performed by FACS AriaII (BD Bioscience). Cell purity was verified as more than 95%. Data were analyzed with the FlowJo software (FlowJo, LLC).

### Quantitative imaging analysis & statistical analysis

The total cell area and the number of the transplanted BM cells were quantified with the image processing software, IMARIS (Bitplane). A 2D object contour iso-surface for each BM cell cluster was created with the Contour Surface function of IMARIS to automatically calculate the area size of each cell cluster. Based on the exported statistical area values of each cluster, the total cell area or the number of clusters categorized for each size of cluster group was analyzed. The average size of the individual BM cells was measured to be 210 ± 81 μm^2^ by *in vitro* observation before the BMT with the same imaging system for the *in vivo* BM imaging. To analyze the formation of the transplanted BM cell cluster over time, the BM cell clusters were categorized into three groups depending on their size: group #1 with a size smaller than 500 μm^2^; group #2 with a size of 500–5,000 μm^2^; and group #3 with a size larger than 5,000 μm^2^. Therefore, group #1 represents a single cell and a group of 2–3 cells. Group #2 represents a small-sized cell cluster comprised of 4–25 cells. Group #3 represents a large cell cluster that includes more than 25 cells. The quantitative analysis of the total cell area in the calvarium BM, sagittal sinus and BM cavity was performed by calculating the proportional ratio of the total cell area to the cropped image area used in the analysis. For statistical analysis, values are presented as the mean standard deviation.

## Results

### Longitudinal nuclear-cytoplasmic visualization of transplanted bone marrow cells

**Fig 1:**
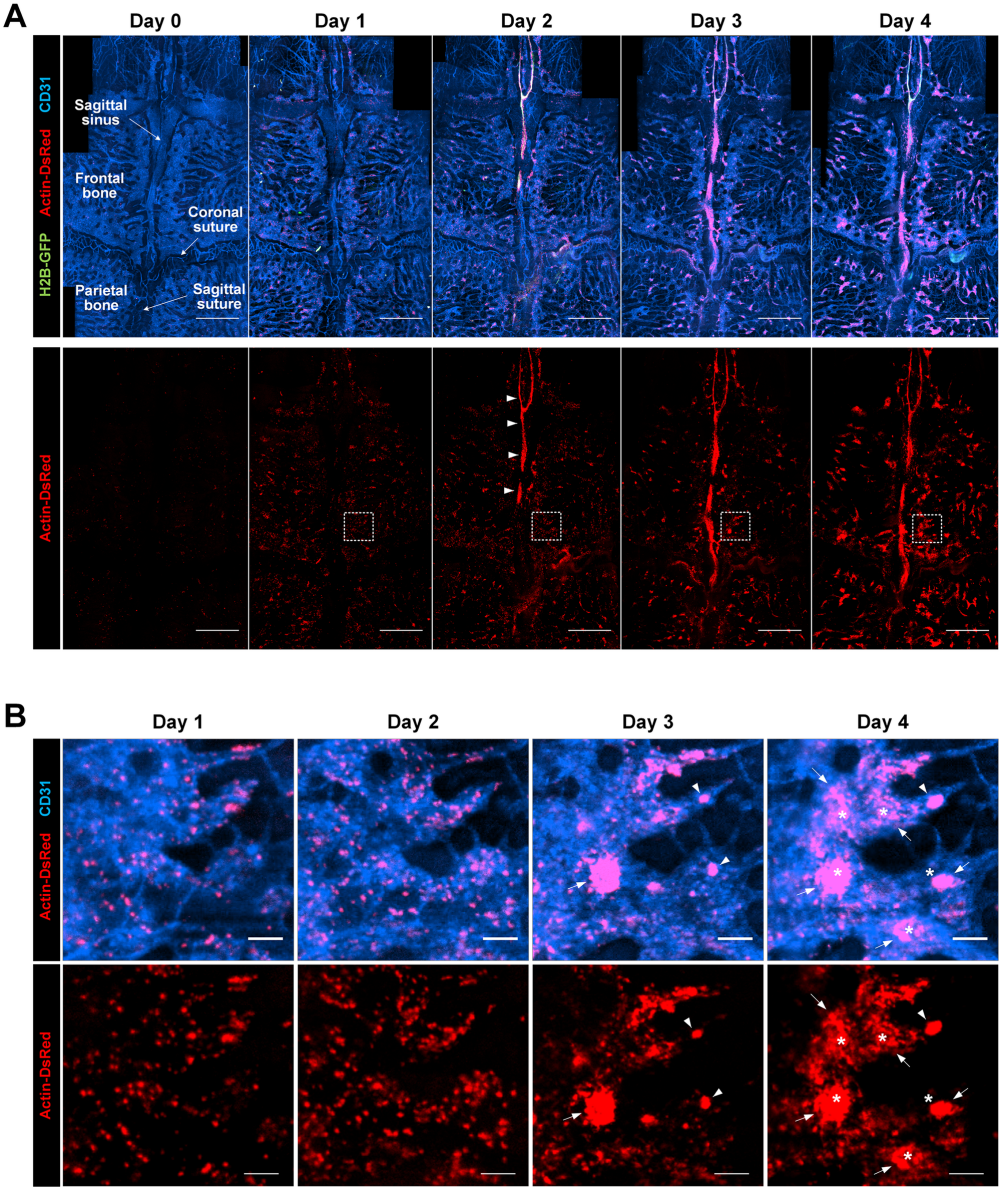
Longitudinal wide-area visualization of the cellular-level behavior of the transplanted BM cells. Mosaic and magnified images longitudinally obtained from the calvarial bone marrow of the recipient mouse *in vivo*. (A) (upper panel) Mosaic images showing the transplanted BM cells (red, actin-DsRed in the cytoplasm and nucleus; green, H2B-GFP in the nucleus) and vascular sinus (blue). (lower panel) Red-color separated mosaic images highlighting the distribution of the transplanted BM cells on each day. (B) Magnified images of (A) at the region marked with dotted squares. The large BM cell clusters with a size larger than 5,000 μm^2^ were marked with an arrow. The small BM cell clusters with a size ranging from 500–5,000 μm^2^ were marked with an arrowhead. The asterisk indicates the BM cell cluster with a significantly increased size at day 4. Scale bars are (A) 1 mm, and (B) 100 μm, respectively.

Using a custom-built laser-scanning confocal platform combined with a stereotaxic mount (S1 Fig), we performed an intravital imaging of transplanted bone marrow cells at the calvarial bone marrow (BM) of the recipient mouse. A subcellular-level nuclear-cytoplasmic morphological visualization of the transplanted BM cells was achieved by utilizing a H2B-GFP/β-actin-DsRed double-transgenic mouse model as a donor mouse for the syngeneic BMT. Wide-area mosaic images of the calvarial BM of the recipient mouse were longitudinally obtained at 1 day intervals after the BMT (Fig 1A). From day 0 to day 1 after the BMT, the transplanted BM cells observed in the calvarial bone marrow were greatly increased in number and sparsely distributed. Interestingly, at day 2, many transplanted BM cells were newly observed around sagittal sinus (arrowhead), while a large number of transplanted BM cells were still sparsely located as seen on day 1. At day 3, the number of sparsely-populated BM cells was decreased. The formation of a large cluster of the transplanted BM cells (arrow) was observed at day 3 as clearly seen in Fig 1B showing the magnified images at the region marked with the dotted squares in Fig 1A. At day 4, the number of large clusters was further increased (arrow).

**Fig 2:**
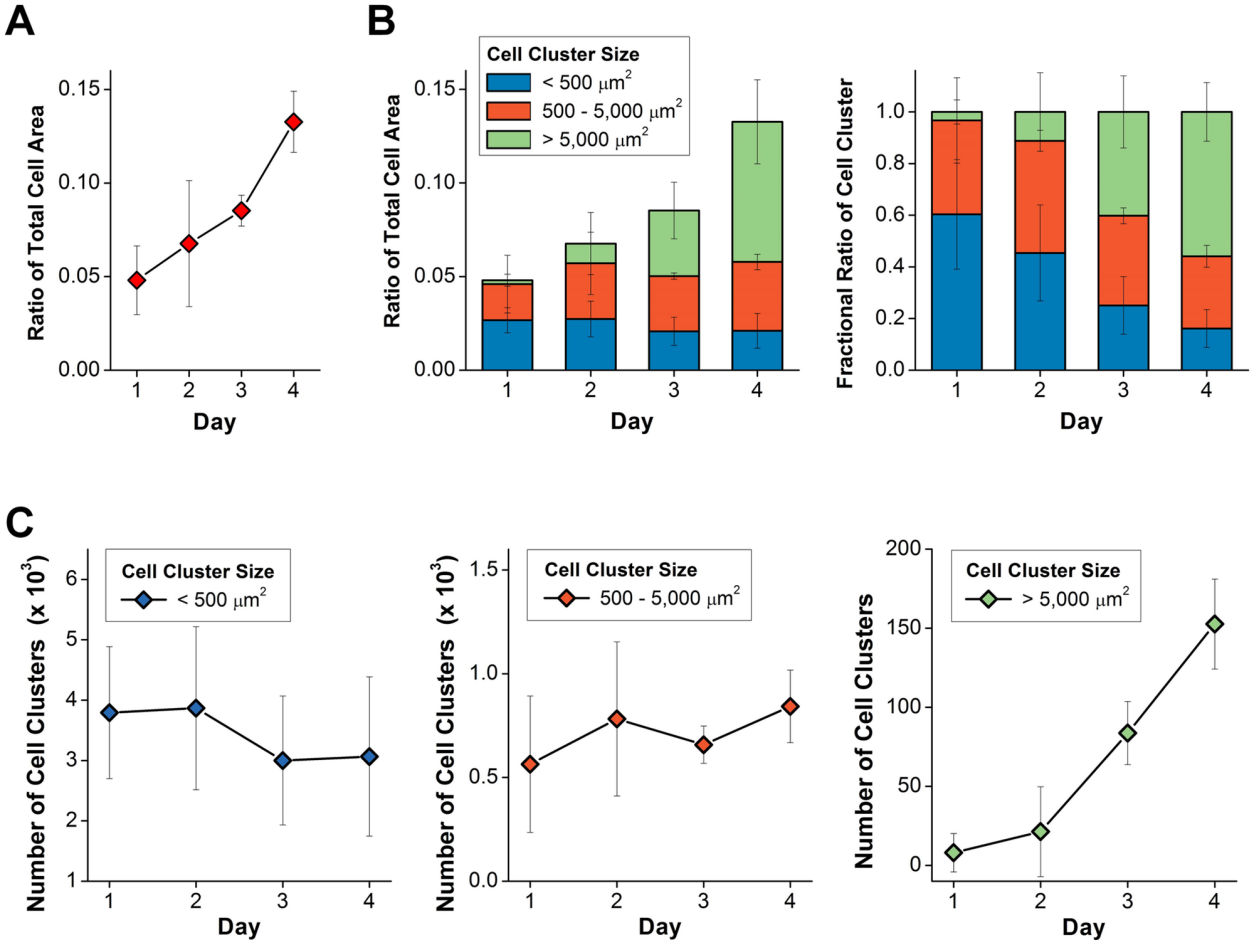
Quantitative imaging analysis of transplanted BM cells. Quantitative imaging analysis was performed by using the large mosaic images of calvarial BM of three BMT mouse models. (A) The ratio of the total area occupied by actin-DsRed expressing cells in the calvalrial BM. (n = 3). (B) The ratio of the total cell area and the fractional ratio occupied by three groups comprising single BM cells or their clusters categorized by their size: < 500 μm^2^; 500–5,000 μm^2^; > 5,000 μm^2^. (C) The number of cell clusters in each group. (n = 3).

Quantitative imaging analysis was performed to identify the behaviors of the transplanted BM cells and their clusters by using the large mosaic images that were obtained independently from three BMT mouse models and cropped so that the similar area and regions are shown in Fig 1A. First, Fig 2A shows the ratio of the total cell area occupied by the actin-DsRed expressing cells in the calvarial BM after the BMT over time. As seen in Fig 2A, the total area of the transplanted BM cell clusters continuously increased until day 4. In Fig 2B and 2C, to analyze the formation of the transplanted BM cell cluster over time, we categorized the BM cell clusters into three groups depending on their size: group #1 with a size smaller than 500 μm^2^; group #2 with a size of 500–5,000 μm^2^; and group #3 with a size larger than 5,000 μm^2^. Fig 2B shows the fractional ratio of each group. Finally, Fig 2C shows the number of cell clusters in each group.

The fractional ratio of the total area occupied by groups #1 and #2 continuously decreased from day 1 to day 4 (Fig 2B). In contrast, the fractional ratio of the total area occupied by group #3 continuously increased over time. Interestingly, the number of cell clusters with a size smaller than 500 μm^2^, group #1, and with a size between 500–5,000 μm^2^, group #2, moderately decreased from day 2 to day 3 and then slightly increased on day 4 (Fig 2C). On the other hand, the number of cell clusters larger than 5,000 μm^2^, group #3, slightly increased from day 1 to day 2 and then rapidly increased from day 2 to day 4. In addition, the distinctive change from day 1 to day 2 is the formation of large transplanted BM cell clusters in sagittal sinus as shown in Fig 1A marked by arrowheads. We performed similar quantitative analyses at different regions in the bone marrow including sagittal sinus and BM cavity as shown in S2A Fig. In sagittal sinus, the ratio of the total cell area in sagittal sinus significantly increased by more than 8 times from day 1 to day 2 (S2B Fig). The total cell area confined in the sagittal sinus continuously increased until day 3 and was maintained at day 4.

**Fig 3:**
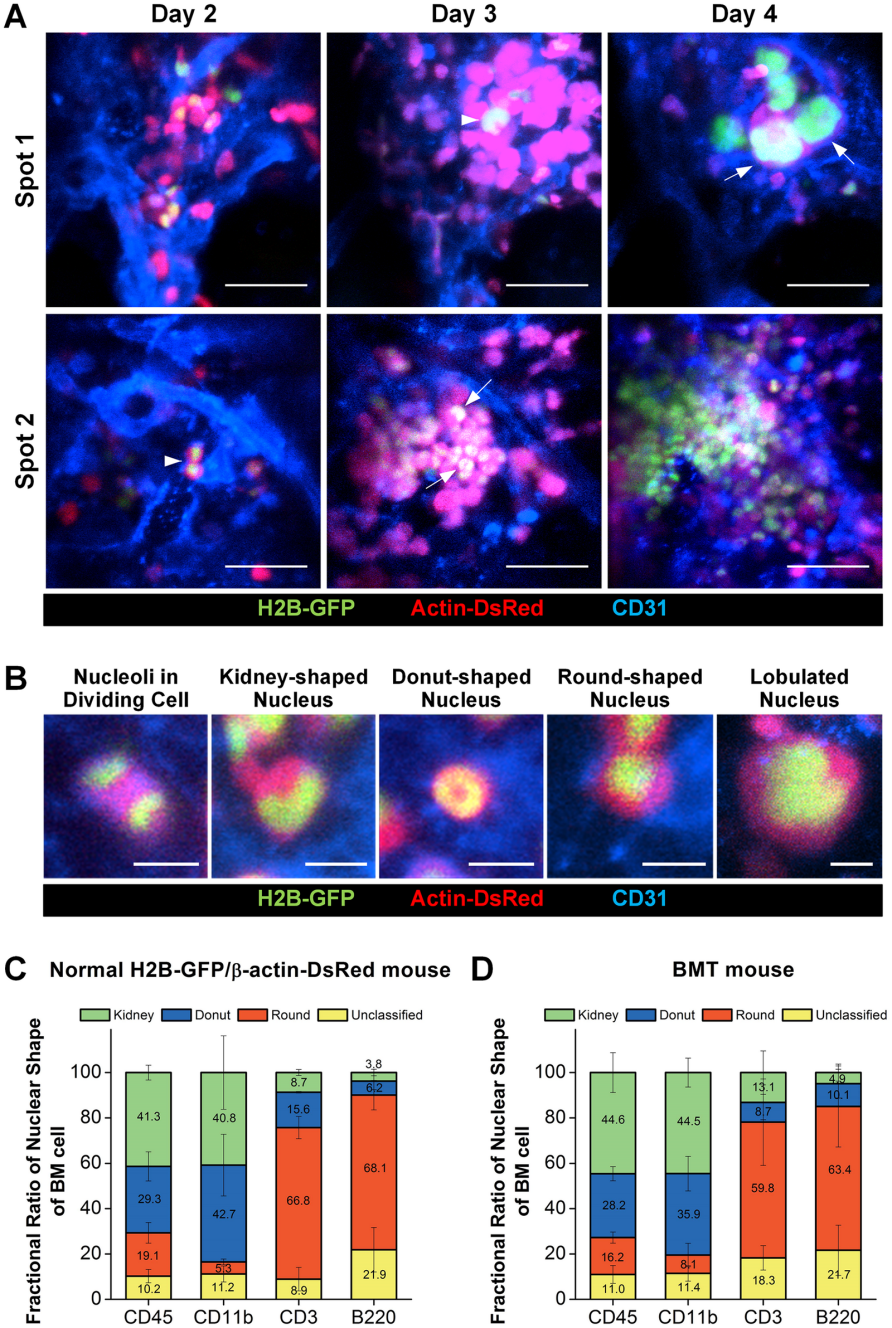
Nuclear-cytoplasmic visualization of the transplanted BM cell. (A) Differentiation of the transplanted bone marrow cells into megakaryocytes (spot 1) and cluster formation (spot 2) were longitudinally observed at sequential time-points *in vivo*. (B) Various shapes of nucleus visualized by nuclear-cytoplasmic dual labeling. (C, D) Fractional ratio of nuclear morphology in (C) the normal BM cells isolated from the BM of H2B-GFP/β-actin-DsRed double-transgenic mouse, (D) the transplanted BM cells expressing H2B-GFP/β-actin-DsRed isolated from the BM of the BMT recipient wildtype mouse (n≥3). Scale bars are (A) 50 μm, and (B) 10 μm, respectively.

Fig 3A shows longitudinal images repetitively obtained *in vivo* at the same area of the calvarial bone marrow after the BMT. A clearly identifiable vascular pattern landmarks enabled the repetitive cellular-level visualization of multiple specific regions in the calvarial bone marrow over several days after the BMT. At spot 1, from day 2 to day 3, a large cluster of the transplanted BM cells expressing DsRed was formed on the upper-right side of the image as previously described in Fig 1B. A promegakaryocyte (arrowhead), an immature megakaryocyte progenitor cell with enlarged nucleus, was observed. At day 4, at the same location, several megakaryocytes (arrow) with a more enlarged and lobulated nucleus were observed. These observations show an active cellular differentiation toward thrombocytes. At spot 2, a couple of transplanted BM cells (arrowhead) with a high nuclear-cytoplasmic ratio were observed at day 2. At day 3, the number of cells greatly increased, which had a kidney- or donut- shaped nucleus (arrow).

Various shapes of nuclei were identifiable as shown in Fig 3B including the following: two nuclei in a dividing cell at telophase and a kidney-shaped nucleus, donut-shaped nucleus, round-shaped nucleus, and large lobulated nucleus. Of note, through repetitive longitudinal imaging of the same region from day 2 to day 4, we identified a promegakaryocyte (arrowhead) and megakaryocytes (arrow) by enlarged nucleus in lobulated shape as shown at spot 1 in Fig 3A. However, except for the megakaryocytes identifiable by their unique lobulated nucleus, the conclusive identification of differentiated cell types requires further validation with more cellular markers in addition to nuclear-cytoplasmic morphological observations. Accordingly, to further explore the possibility of identifying the lineage or type of differentiating cell after the BMT by the nuclear-cytoplasmic morphology visualization, we performed imaging analysis of the BM cells with various molecular markers specific for differentiated cell types. We sorted the BM cells of the BMT host by FACS according to various cell lineage specific markers (CD45, whole hematopoietic cells; CD11b, myeloid cell lineage; CD3, T lymphocyte, and B220, B lymphocyte) and then identified the nuclear morphology of the sorted cells *in vitro* by fluorescence confocal microscope. We observed the distinguishable morphological characteristics of nucleus such as the donut-shape, kidney-shape and round-shape in cells differentiating to each cell lineage. Representative images of each distinct nuclear shape are shown in S3 Fig.

To verify the distinct nuclear morphology in both the normal BM cells and the transplanted BM cells according to each cell type, we performed *in vitro* imaging in both groups. First, as shown in Fig 3C, whole CD45^+^ BM cells isolated from the normal BM of the H2B-GFP/β-actin-DsRed double-transgenic mouse exhibited a balanced proportion of different nuclear morphologies: round-shape (19.1%), donut-shape (29.3%) and kidney-shape (41.3%). Most of the CD11b^+^ myeloid cells had either a donut- or kidney- shaped nucleus (42.7% and 40.8% each). In contrast, the majority of CD3^+^ T lymphocytes and B220^+^ B lymphocytes, lymphoid lineage cells, had a round-shaped nucleus (66.8% and 68.1% each).

Second, as shown in Fig 3D, the transplanted BM cells expressing H2B-GFP/β-actin-DsRed isolated from the BM of the recipient wildtype mouse at 14 days after the BMT showed similar results as that of the wildtype mouse. Whole CD45^+^ BM cells exhibited a similar composition of nuclear morphologies, round-shape (16.2%), donut-shape (28.2%) and kidney-shape (44.6%), as shown in Fig 3D. The majority of CD11b^+^ myeloid cells had either a donut- or kidney-shaped nucleus (35.9% and 44.5% each). And most of the CD3^+^ T lymphocytes and B220^+^ B lymphocytes had a round shaped nucleus (59.8% and 63.4% each).

To summarize, more than 80% of the DsRed expressing CD11^+^ myeloid lineage cells in the BM of both the normal donor mouse and BMT recipient mouse had either of a donut- or kidney-shaped nucleus. Additionally, more than 60% of the DsRed expressing CD3^+^ or B220^+^ lymphoid lineage cells had a round-shaped nucleus. Therefore, the increasing number of cells with a donut- or kidney-shaped nucleus observed in Fig 3A at day 3 and 4 might mostly be CD11b^+^ myeloid cells suggesting myeloid cell differentiation.

### Dynamic nuclear-cytoplasmic visualization for proliferation monitoring

**Fig 4:**
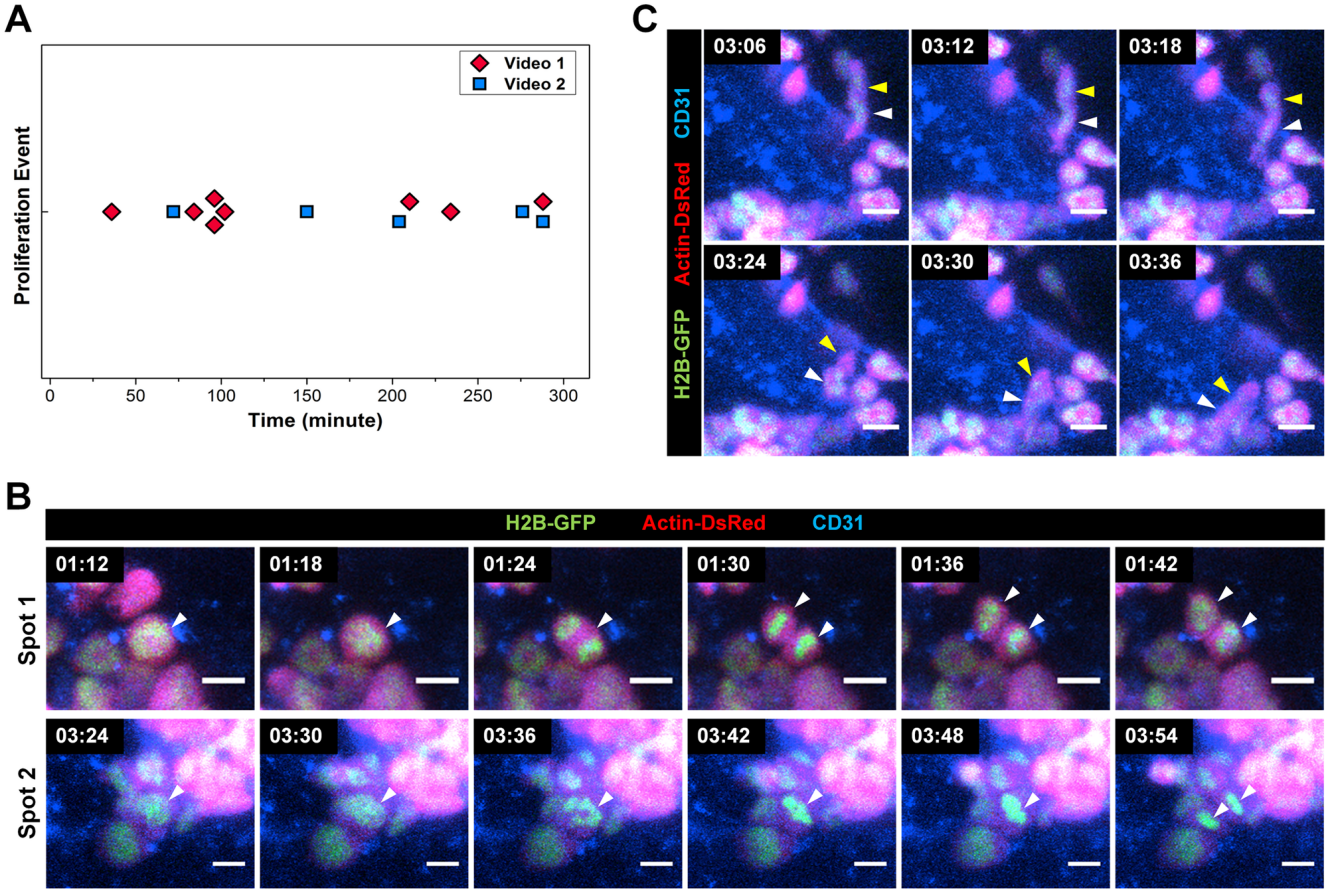
Dynamic time-lapse nuclear-cytoplasmic visualization of the transplanted BM cell. Multi-position time-lapse imaging of the calvarial bone marrow at 6 minute intervals was performed 3 days after the BMT. (A) Proliferation events observed during the 5 hour time-lapse imaging (S1 and S2 Movies). (B, C) Representative time-lapse image sequences showing (B) proliferation (S3 and S4 Movies) or (C) migration of the transplanted BM cell (S5 Movie). Scale bar is 10 μm.

Multi-position time-lapse imaging of the calvarial bone marrow at 6 minute intervals was performed to dynamically monitor the transplanted bone marrow cells at day 3 when the cluster of the transplanted BM cells was observed (S1 and S2 Movies). Fig 4A shows all of the proliferation events observed during the 5-hour time-lapse imaging at each imaging spots. Fig 4B shows the magnified time-lapse image sequences showing the subcellular-level nuclear chromosome dynamics during the entire cell division process; nucleus alignment in a rectangular shape starting at 1:12 and 3:24 (hour:minute), nuclear fission, cytokinesis, and division into two independent cells at 1:30 and 3:54, respectively (S3 and S4 Movies). In Fig 4C, we could identify two transplanted BM cells at the perivascular area which are marked by white and yellow arrowheads, respectively. They entered into the vascular sinus and migrated in a collective manner toward the area where many other transplanted BM cells were clustered (S5 Movie). These image sequences and videos obtained clearly show that the transplanted BM cells are highly dynamic at day 3 after the BMT: they actively proliferate, migrate in the bone marrow vascular sinus, and eventually differentiate into various cell types.

## Discussion

We performed a quantitative imaging analysis using large mosaic images repeatedly obtained from the same mouse over 4 days after a BMT (n = 3). It revealed the detailed collective behaviors of the transplanted BM cells which were sequentially observed in sagittal sinus and the BM cavity after the BMT. At day 1, the transplanted BM cells homed to the recipient’s BM and were mostly distributed in the BM cavity as either of single cells or small clusters (Figs 1A and 1B and 2B). At day 2, the accumulation of the transplanted BM were observed at sagittal sinus as shown in Fig 1A, which continued until day 3 then slowed down at day 4 (S2B Fig). In contrast, in the BM cavity, the total cell area of transplanted BM continuously and rapidly increased until day 4 (Fig 2A). In addition, in the BM cavity, the active formation of large BM cell clusters was observed at a later time point, day 3 and 4 (Fig 1A and 1B). Interestingly, the fractional ratio of total area occupied by groups #1 and #2 continuously decreased from day 1 to day 4 while the fractional ratio of the total area occupied by group #3 continuously increased until day 4 (Fig 2B). The decrease of the area and the number of BM cell clusters categorized as group #1 and #2 and the appearance of large BM cell clusters (arrow) from day 2 to day 3 (Fig 1B) might be the result of the redistribution of the transplanted BM cells to specific sites or the disappearance of transplanted BM cells that failed to engraft. In addition, during the following period from day 3 to day 4, a significant increase in the area and number of large BM cell clusters categorized as group #3 was observed suggesting rapid proliferation at specific sites such as the locations marked by an asterisk in Fig 1B.

In addition, we newly demonstrated subcellular-level nuclear-cytoplasmic morphological visualization of transplanted bone marrow cells with an H2B-GFP/β-actin-DsRed double-transgenic mouse model as the donor of the BMT. It enabled a detailed *in vivo* imaging analysis of post-transplantation cellular-level behaviors of the transplanted BM cells including cluster formation, migration, active proliferation, and differentiation into certain cell types [39–42]. Nuclear-cytoplasmic dual labeling has a clear advantage helping to understand cell behaviors that occur in densely populated cell clusters where every cell is contacting each other as shown in Fig 4B and 4C because it enables the identification of individual cells by their nucleus. The intravital imaging experiment with cytoplasmic labeling alone has limitations in such conditions because it is hard to discriminate a single bone marrow cell in the cell cluster. Moreover, because the transplanted bone marrow cells have a large diversity in cell body size and morphology depending on the cell types as presented in Fig 3, it would not be sufficient with only cytoplasmic labeling to optically observe the various dynamic behavior of individual transplanted BM cells in detail. As the bone marrow cells have various nuclear morphologies for differential cell identities such as donut-shape, kidney-shape, round-shape and lobulated-shape, the nuclear-cytoplasmic dual labeling would be more appropriate for discriminating the behavior of an individual BM cell from others nearby and monitoring the *in vivo* proliferation, differentiation, and dynamic movement of each BM cell as presented in Figs 3 and 4. In addition, based on fluorescent visualization of the nuclear morphology *in vitro*, we could identify the cellular lineages of the transplanted BM cells. Nuclear morphology can be discriminated using our methodology, what allows to track major BM cell lineages such as myeloid and lymphoid.

Therefore, *in vivo* visualization of the morphological shape of the nucleus of the transplanted BM cells could be a useful method to perform the *in vivo* longitudinal monitoring of differentiation, especially into CD11b^+^ myeloid cells, CD3^+^ T lymphoid or B220^+^ B lymphoid cells and megakaryocytes identifiable by their distinct nuclear morphology, and proliferation of the transplanted BM cells in the recipient BM. Furthermore, this nuclear-cytoplasmic imaging method can be combined with other fluorescent labeling techniques based on an exogenous fluorophore conjugated with various targeting moieties including antibodies, aptamers, peptides or small molecules, thereby further improving the specificity in the monitoring of transplanted BM cell differentiation.

The intensity of H2B-GFP expression in the nucleus was not consistent between the transplanted BM cells which could be improved by a further purification procedure based on fluorescence intensity such as fluorescence activated cell sorting (FACS). Transplanted c-kit expressing BM cells were enriched with not only hematopoietic stem cells but also hematopoietic progenitor cells such as CMP, MEP, and GMP. Therefore, most of the specific sites where the transplanted BM cells migrated and rapidly proliferated might not be exclusively a hematopoietic stem cell niche. However, it may be more closely related to the clinical situation of a BMT than transplanting only purified HSCs. Additionally, it has been shown that co-transplantation of hematopoietic progenitor cells such as CMP and GMP has clinical potential in accelerating immune reconstitution after BMT without post-transplant complications (e.g., graft versus host disease (GvHD) and viral infections) [43, 44]. Vascular remodeling after extensive irradiation therapy was previously reported which can preclude using the vasculature as a landmark for repetitive imaging of the same sites [45, 46]. However, the degree of vascular remodeling after sub-lethal irradiation with a dose of 6 Gy was relatively not significant and observed only in some capillaries [46]. As a result, by using the stereotaxic mount in conjunction with the fluorescent-labeled vasculature landmark, the same area in the calvarial bone marrow was easily identified in repetitive imaging sessions over several days.

We believe that the nuclear-cytoplasmic morphological imaging strategy of transplanted bone marrow cells can be a useful method to directly monitor proliferation and further investigate underlying molecular and cellular mechanisms [47–49] involved in BMT to alleviate various pathological conditions such as leukemia, anemia, lymphoma or related complications such as GvHD. Specifically, for example, by using a genetically modified mice including knock-out mice for various disease related genes or fluorescent-reporter mice for specific cells such as vascular endothelial cells, or bone marrow resident immune/stromal cells as a recipient for the BMT, the composition of the cellular microenvironment or cell-to-cell interactions affecting the efficacy of the BM cell engraftment, proliferation and differentiation could be investigated by the proposed *in vivo* nuclear-cytoplasmic visualization.

## Supporting information

S1 Fig Intravital imaging setup. (A) Schematic of the custom-built confocal microscopy system: ND, neutral density filter; DBS, dichroic beam splitter; BPF, bandpass filter; M, mirror; PMT, photomultiplier tube; OBJ, objective lens. (B) Photograph of the stereotaxic instrument comprised of ear bars, probe holders and a mouth adapter with a U-shape holder for the cover glass. (C) Photograph of the assembled stereotaxic set-up on the motorized XYZ translation stage, and the mouse cranium exposed for in vivo imaging (red dotted-line square).
(TIF)

S2 Fig Quantitative analysis of transplanted BM cells at the sagittal sinus and BM cavity. (A) Illustration of the region of sagittal sinus and BM cavity in calvarium. (B) Total area occupied by actin-DsRed expressing cell in the sagittal sinus.
(TIF)

S3 Fig Representative in vitro visualization of distinctive nuclear morphology of specific cell types from normal H2B-GFP/β-actin-DsRed double-transgenic mouse BM cells. Representative magnified images of CD11b^+^ cells with donut and kidney shape of nucleus. Representative magnified images of CD3^+^ cell and B220^+^ cell with round shape of nucleus. Scale bar is 10μm.
(TIF)

S1 Movie Dynamic nuclear-cytoplasmic visualization of the transplanted bone marrow cells. Time-lapse imaging of the calvarial bone marrow at 6 minutes interval was performed for 5 hours at 3 days after the bone marrow transplantation. Proliferation (white arrow) and migration (yellow arrow) of the transplanted bone marrow cells were visible. All of 8 proliferation events are listed in Fig 4A. Scale bar is 50 μm.
(MOV)

S2 Movie Dynamic nuclear-cytoplasmic visualization of the transplanted bone marrow cells. Time-lapse imaging of the calvarial bone marrow at 6 minutes interval was performed for 5 hours at 3 days after the bone marrow transplantation. Proliferation (white arrow) of the transplanted bone marrow cells was visible. All of 5 proliferation events are listed in Fig 4A. Scale bar is 50 μm.
(MOV)

S3 Movie Proliferation of the transplanted bone marrow cells. Scale bar is 10 μm.
(MOV)

S4 Movie Proliferation of the transplanted bone marrow cells. Scale bar is 10 μm.
(MOV)

S5 Movie Migration of the transplanted bone marrow cells. Scale bar is 10 μm.
(MOV)
