# Supplementary figures and images for: Intravital longitudinal wide-area imaging of dynamic bone marrow engraftment and multilineage differentiation through nuclear-cytoplasmic labeling
Source: PLoS One. 2017 Nov 3;12(11):e0187660. doi: 10.1371/journal.pone.0187660 (PMC5669471; doi:10.1371/journal.pone.0187660)

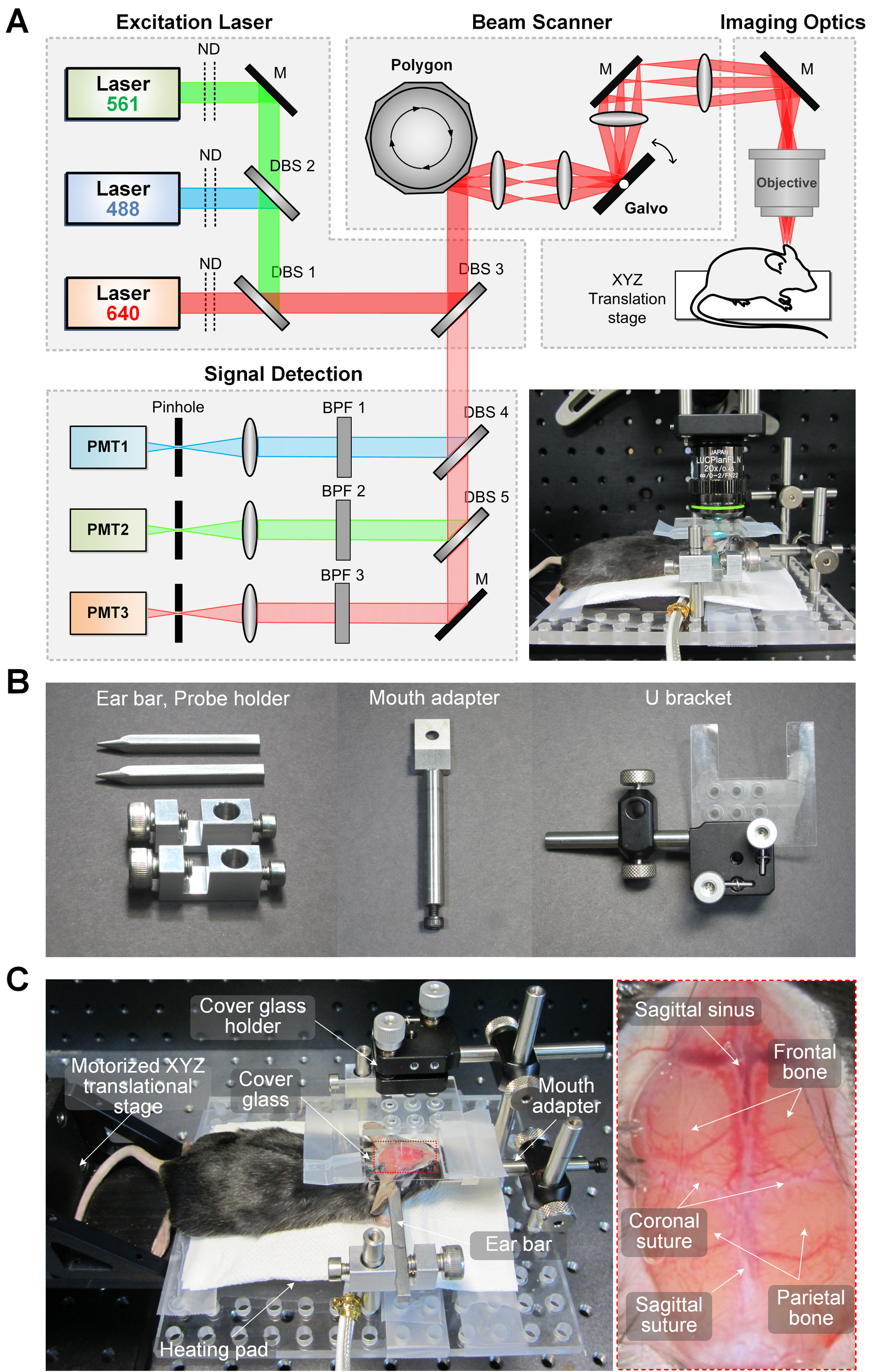

Supplement: S1 Fig — (A) Schematic of the custom-built confocal microscopy system: ND, neutral density filter; DBS, dichroic beam splitter; BPF, bandpass filter; M, mirror; PMT, photomultiplier tube; OBJ, objective lens. (B) Photograph of the stereotaxic instrument comprised of ear bars, probe holders and a mouth adapter with a U-shape holder for the cover glass. (C) Photograph of the assembled stereotaxic set-up on the motorized XYZ translation stage, and the mouse cranium exposed for in vivo imaging (red dotted-line square). (TIF) [file pone.0187660.s001.tif]

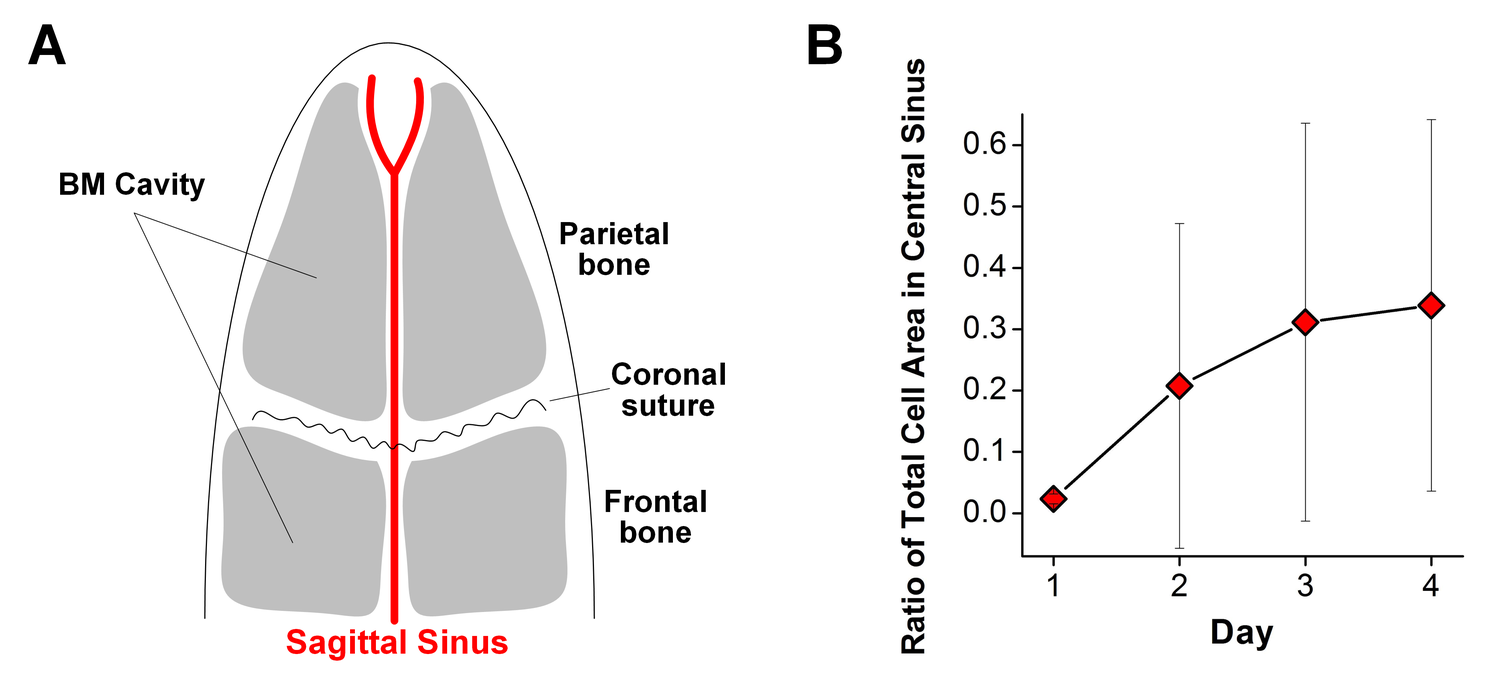

Supplement: S2 Fig — (A) Illustration of the region of sagittal sinus and BM cavity in calvarium. (B) Total area occupied by actin-DsRed expressing cell in the sagittal sinus. (TIF) [file pone.0187660.s002.tif]

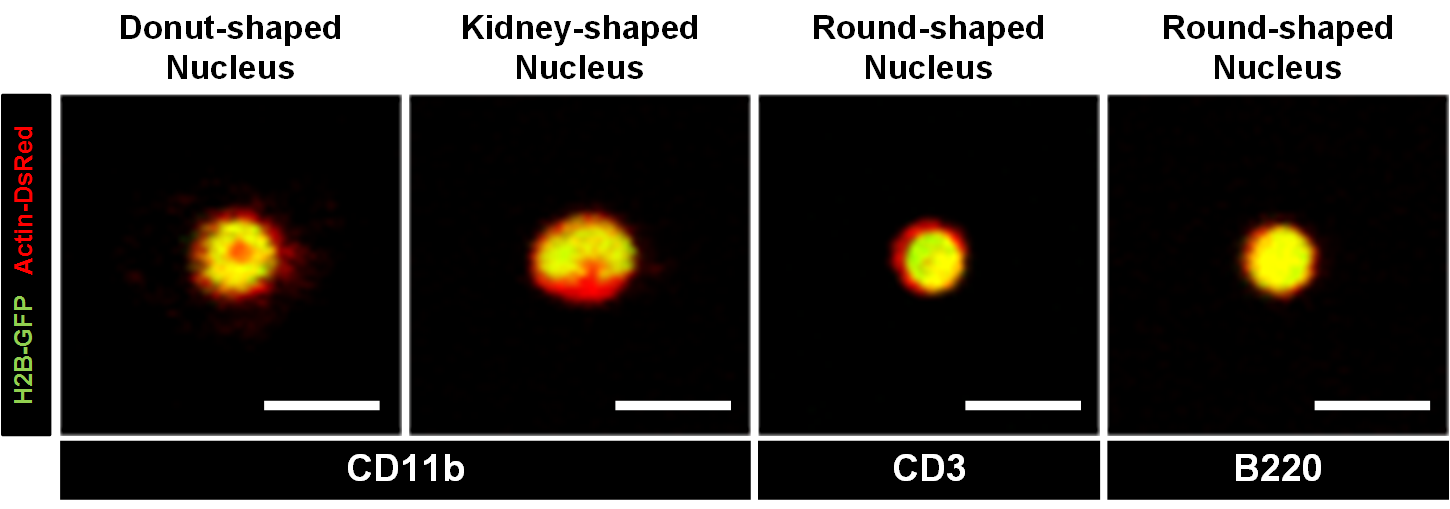

Supplement: S3 Fig — Representative magnified images of CD11b+ cells with donut and kidney shape of nucleus. Representative magnified images of CD3+ cell and B220+ cell with round shape of nucleus. Scale bar is 10μm. (TIF) [file pone.0187660.s003.tif]
